# Redetermination of *catena*-poly[[chlorido­(thio­urea-κ*S*)copper(I)]-μ-thio­urea-κ^2^ *S*:*S*] at 100 K

**DOI:** 10.1107/S1600536812004448

**Published:** 2012-02-10

**Authors:** Hafid Zouihri

**Affiliations:** a Laboratoire Privé de Cristallographie (LPC), Kénitra, Morocco.

## Abstract

The structure of the polymeric title compound, [CuCl(CH_4_N_2_S)_2_]_*n*_, has been redetermined to modern standards of precision with anisotropic refinement and location of the H atoms. The previous structure report [Spofford & Amma (1970 ▶). *Acta Cryst.* B**26**, 1474–1483] is generally confirmed to higher precision [typical Cu—S bond length s.u. values = 0.005 (old) and 0.001 Å (new)]. The asymmetric unit contains two formula units, with both Cu^I^ atoms coordinated by one terminal S atom and two bridging S atoms of thio­urea ligands. This connectivity leads to polymeric [100] chains in the crystal. If very long contacts to nearby chloride ions [2.8687 (9) and 3.1394 (12) Å] are considered to be bonding, then very distorted CuS_3_Cl tetra­hedral coordination polyhedra arise. The crystal structure is consolidated by weak intra- and inter-chain N—H⋯S and N—H⋯Cl hydrogen bonds.

## Related literature

For the structure of a related thio­urea salt, see: Zouihri (2012 ▶). For the previous structure determination of the title compound, see: Spofford & Amma (1970 ▶).
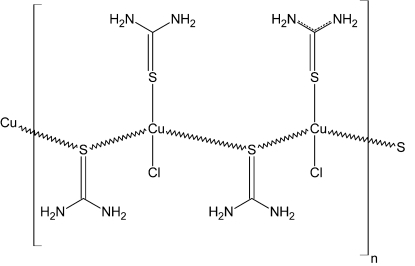


## Experimental

### 

#### Crystal data

[CuCl(CH_4_N_2_S)_2_]
*M* *_r_* = 251.24
Monoclinic, 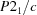

*a* = 5.8043 (2) Å
*b* = 8.1292 (3) Å
*c* = 35.9657 (12) Å
β = 92.326 (2)°
*V* = 1695.62 (10) Å^3^
*Z* = 8
Mo *K*α radiation
μ = 3.32 mm^−1^
*T* = 100 K
0.45 × 0.18 × 0.07 mm

#### Data collection

Bruker APEXII CCD detector diffractometer
Absorption correction: multi-scan (*SADABS*; Sheldrick, 1996 ▶) *T*_min_ = 0.493, *T*_max_ = 0.793
18106 measured reflections
4089 independent reflections
3372 reflections with *I* > 2σ(*I*)
*R*_int_ = 0.040

#### Refinement

*R*[*F* ^2^ > 2σ(*F* ^2^)] = 0.043
*wR*(*F* ^2^) = 0.081
*S* = 1.15
4089 reflections
245 parameters
16 restraints
All H-atom parameters refined
Δρ_max_ = 0.58 e Å^−3^
Δρ_min_ = −0.85 e Å^−3^

### 

Data collection: *APEX2* (Bruker, 2005 ▶); cell refinement: *SAINT* (Bruker, 2005 ▶); data reduction: *SAINT*; program(s) used to solve structure: *SHELXS97* (Sheldrick, 2008 ▶); program(s) used to refine structure: *SHELXL97* (Sheldrick, 2008 ▶); molecular graphics: *PLATON* (Spek, 2009 ▶); software used to prepare material for publication: *publCIF* (Westrip, 2010 ▶).

## Supplementary Material

Crystal structure: contains datablock(s) I, global. DOI: 10.1107/S1600536812004448/hb6598sup1.cif

Structure factors: contains datablock(s) I. DOI: 10.1107/S1600536812004448/hb6598Isup2.hkl

Additional supplementary materials:  crystallographic information; 3D view; checkCIF report

## Figures and Tables

**Table 1:** Selected bond lengths (Å)

Cu1—S1	2.3091 (9)
Cu1—S2	2.2617 (10)
Cu1—S3	2.2728 (10)
Cu1—Cl2	3.1394 (12)
Cu2—S1^i^	2.3081 (9)
Cu2—S3	2.2747 (9)
Cu2—S4	2.2421 (9)
Cu2—Cl1	2.8687 (9)

Symmetry code: (i) 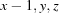.

**Table 2:** Hydrogen-bond geometry (Å, °)

*D*—H⋯*A*	*D*—H	H⋯*A*	*D*⋯*A*	*D*—H⋯*A*
N1—H1*A*⋯Cl1^ii^	0.85 (3)	2.44 (3)	3.230 (3)	154 (4)
N1—H1*B*⋯S4^iii^	0.87 (3)	2.55 (3)	3.404 (4)	171 (3)
N2—H2*A*⋯S2	0.87 (3)	2.54 (3)	3.379 (3)	164 (3)
N2—H2*B*⋯Cl1^ii^	0.86 (3)	2.56 (3)	3.344 (3)	153 (3)
N3—H3*A*⋯S1	0.87 (3)	2.65 (3)	3.488 (4)	164 (3)
N3—H3*B*⋯Cl2^iv^	0.86 (4)	2.57 (4)	3.373 (4)	156 (4)
N4—H4*A*⋯Cl2^iv^	0.85 (3)	2.49 (3)	3.309 (3)	161 (3)
N4—H4*B*⋯Cl2^v^	0.84 (3)	2.55 (3)	3.340 (3)	157 (4)
N5—H5*A*⋯Cl2	0.86 (3)	2.35 (3)	3.197 (3)	167 (4)
N5—H5*B*⋯Cl2^vi^	0.86 (3)	2.55 (4)	3.356 (3)	157 (3)
N6—H6*A*⋯Cl2^i^	0.87 (4)	2.66 (4)	3.323 (3)	134 (4)
N6—H6*B*⋯Cl1	0.86 (2)	2.44 (2)	3.296 (3)	174 (3)
N7—H7*A*⋯Cl1	0.85 (3)	2.61 (4)	3.343 (4)	145 (3)
N7—H7*B*⋯Cl1^vii^	0.87 (4)	2.55 (4)	3.367 (4)	157 (3)
N8—H8*A*⋯Cl1^vii^	0.86 (4)	2.54 (4)	3.326 (4)	152 (3)
N8—H8*B*⋯Cl1^viii^	0.85 (3)	2.55 (3)	3.298 (4)	148 (4)

Symmetry codes: (i) 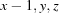; (ii) 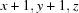; (iii) 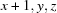; (iv) 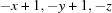; (v) 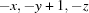; (vi) 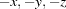; (vii) 
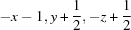
; (viii) 
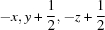
.

**Fig. 1.**
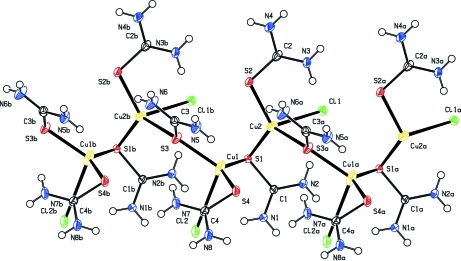
Molecular view of the title compound showing displacement ellipsoids drawn at the 50% probability level.

**Fig. 2.**
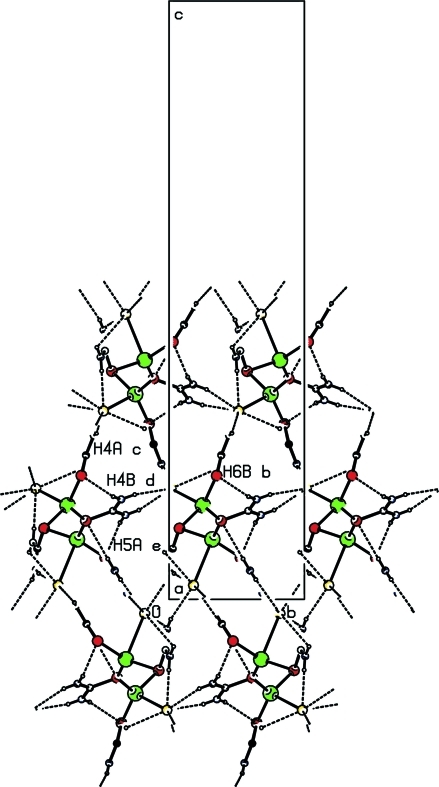
Projection of the title compound along the a axis, H-bonds are represented by dashed lines.

## supplementary crystallographic information

### Comment

In a former paper, we reported the crystal structure of (Diaminomethylidene)sulfonium chloride-thiourea (3/2) [Zouihri, 2012]. In this paper, we report the synthesis and the structure of the title compound. It was previously described by Spofford & Amma (1970).

The asymmetric unit of the title compound is shown in Fig. 1. The Cu^I^ ions have distorted tetrahedral coordination geometries formed by two bridging thiourea ligands, one terminal thiourea ligand and one chloride ion (Cu—S and Cu—Cl distances in the range of 2.2618 (10) Å to 3.1392 (12) Å) generating parallel one-dimensional polymeric chains propagating in the *a* axis direction.

In the crystal structure, there are two different types of hydrogen bonds (Table 1, Fig. 2). Intra-chain N—H···S and N—H···Cl interactions appear to influence the conformation of the helical chains while inter-chain N—H···S and N—H···Cl interactions crosslink the chains.

### Experimental

2 mmol of CuCl_2_ and 1 mmol of (Diaminomethylidene)sulfonium chloride-thiourea (3/2) [Zouihri, 2012] in 5 ml of ethanol were refluxed for 1 h, forming a colorless solution. The solution was allowed to evaporate slowly and colourless prisms were obtained after several days.

### Refinement

All H atoms were located from difference Fourier maps and refined isotrpically, with restained distance N—H = 0.86 (2) A.

### Crystal data

**Table:**

[CuCl(CH_4_N_2_S)_2_]	*F*(000) = 1008
*M**_r_* = 251.24	*D*_x_ = 1.968 Mg m^−^^3^
Monoclinic, *P*2_1_/*c*	Mo *K*α radiation, λ = 0.71073 Å
Hall symbol: -P 2ybc	Cell parameters from 368 reflections
*a* = 5.8043 (2) Å	θ = 1.7–27.2°
*b* = 8.1292 (3) Å	µ = 3.32 mm^−^^1^
*c* = 35.9657 (12) Å	*T* = 100 K
β = 92.326 (2)°	Prism, colourless
*V* = 1695.62 (10) Å^3^	0.45 × 0.18 × 0.07 mm
*Z* = 8	

### Data collection

**Table:**

Bruker APEXII CCD detector diffractometer	4089 independent reflections
Radiation source: fine-focus sealed tube	3372 reflections with *I* > 2σ(*I*)
Graphite monochromator	*R*_int_ = 0.040
ω and φ scans	θ_max_ = 28.0°, θ_min_ = 2.6°
Absorption correction: multi-scan (*SADABS*; Sheldrick, 1996)	*h* = −7→7
*T*_min_ = 0.493, *T*_max_ = 0.793	*k* = −10→10
18106 measured reflections	*l* = −47→33

### Refinement

**Table:**

Refinement on *F*^2^	Primary atom site location: structure-invariant direct methods
Least-squares matrix: full	Secondary atom site location: difference Fourier map
*R*[*F*^2^ > 2σ(*F*^2^)] = 0.043	Hydrogen site location: inferred from neighbouring sites
*wR*(*F*^2^) = 0.081	All H-atom parameters refined
*S* = 1.15	*w* = 1/[σ^2^(*F*_o_^2^) + (0.0226*P*)^2^ + 3.6977*P*] where *P* = (*F*_o_^2^ + 2*F*_c_^2^)/3
4089 reflections	(Δ/σ)_max_ = 0.001
245 parameters	Δρ_max_ = 0.58 e Å^−^^3^
16 restraints	Δρ_min_ = −0.85 e Å^−^^3^

### Special details

**Table:**

Geometry. All e.s.d.'s (except the e.s.d. in the dihedral angle between two l.s. planes) are estimated using the full covariance matrix. The cell e.s.d.'s are taken into account individually in the estimation of e.s.d.'s in distances, angles and torsion angles; correlations between e.s.d.'s in cell parameters are only used when they are defined by crystal symmetry. An approximate (isotropic) treatment of cell e.s.d.'s is used for estimating e.s.d.'s involving l.s. planes.
Refinement. Refinement of *F*^2^ against ALL reflections. The weighted *R*-factor *wR* and goodness of fit *S* are based on *F*^2^, conventional *R*-factors *R* are based on *F*, with *F* set to zero for negative *F*^2^. The threshold expression of *F*^2^ > σ(*F*^2^) is used only for calculating *R*-factors(gt) *etc*. and is not relevant to the choice of reflections for refinement. *R*-factors based on *F*^2^ are statistically about twice as large as those based on *F*, and *R*- factors based on ALL data will be even larger.

### Fractional atomic coordinates and isotropic or equivalent isotropic displacement parameters (Å^2^)

**Table:**

	*x*	*y*	*z*	*U*_iso_*/*U*_eq_	
Cu1	0.20578 (8)	0.31958 (6)	0.100266 (15)	0.02808 (13)	
Cu2	−0.13231 (8)	0.24956 (6)	0.157138 (12)	0.02293 (12)	
Cl1	−0.45164 (14)	0.01524 (10)	0.18514 (2)	0.01749 (17)	
Cl2	0.35733 (14)	0.18063 (12)	0.02357 (3)	0.0253 (2)	
S3	0.05347 (13)	0.07669 (10)	0.11866 (2)	0.01369 (17)	
S4	0.06418 (14)	0.35188 (12)	0.20688 (3)	0.0207 (2)	
S2	0.02223 (14)	0.52774 (12)	0.06966 (3)	0.01974 (19)	
S1	0.55003 (14)	0.38438 (11)	0.13126 (3)	0.01895 (19)	
C4	−0.1303 (6)	0.3857 (4)	0.24079 (10)	0.0203 (8)	
C3	−0.1615 (5)	0.0262 (4)	0.08459 (9)	0.0148 (7)	
C1	0.5411 (6)	0.5852 (4)	0.14784 (10)	0.0183 (7)	
C2	0.2234 (6)	0.6102 (4)	0.04098 (10)	0.0179 (7)	
N3	0.4489 (5)	0.5983 (5)	0.04812 (10)	0.0268 (8)	
N1	0.7193 (6)	0.6469 (4)	0.16698 (10)	0.0277 (8)	
N7	−0.3535 (5)	0.3549 (5)	0.23575 (10)	0.0298 (8)	
N2	0.3566 (5)	0.6769 (4)	0.14099 (9)	0.0224 (7)	
N4	0.1543 (5)	0.6897 (5)	0.01073 (9)	0.0259 (7)	
N6	−0.3721 (5)	−0.0059 (4)	0.09504 (9)	0.0227 (7)	
N5	−0.1090 (5)	0.0156 (4)	0.04974 (8)	0.0218 (7)	
N8	−0.0560 (6)	0.4419 (5)	0.27368 (10)	0.0295 (8)	
H5A	0.026 (4)	0.043 (5)	0.0426 (12)	0.037 (13)*	
H6A	−0.482 (6)	−0.015 (6)	0.0781 (11)	0.049 (15)*	
H7A	−0.398 (7)	0.304 (5)	0.2160 (8)	0.034 (13)*	
H8A	−0.148 (6)	0.478 (6)	0.2898 (10)	0.040 (14)*	
H3A	0.500 (7)	0.538 (4)	0.0666 (8)	0.023 (11)*	
H4A	0.258 (5)	0.731 (5)	−0.0024 (10)	0.024 (11)*	
H5B	−0.207 (6)	−0.017 (5)	0.0328 (9)	0.028 (11)*	
H6B	−0.401 (7)	0.005 (5)	0.1181 (6)	0.027 (12)*	
H7B	−0.443 (7)	0.391 (6)	0.2526 (10)	0.043 (14)*	
H8B	0.082 (4)	0.477 (6)	0.2753 (14)	0.048 (15)*	
H2B	0.358 (7)	0.775 (3)	0.1496 (11)	0.025 (12)*	
H1B	0.817 (6)	0.573 (4)	0.1748 (11)	0.025 (11)*	
H4B	0.016 (4)	0.692 (5)	0.0030 (12)	0.030 (12)*	
H3B	0.537 (7)	0.639 (6)	0.0320 (10)	0.041 (14)*	
H2A	0.249 (5)	0.639 (5)	0.1260 (9)	0.026 (11)*	
H1A	0.715 (8)	0.743 (3)	0.1764 (13)	0.044 (14)*	

### Atomic displacement parameters (Å^2^)

**Table:**

	*U*^11^	*U*^22^	*U*^33^	*U*^12^	*U*^13^	*U*^23^
Cu1	0.0184 (2)	0.0208 (2)	0.0442 (3)	−0.00450 (19)	−0.0103 (2)	0.0088 (2)
Cu2	0.0198 (2)	0.0286 (3)	0.0199 (2)	0.00834 (19)	−0.00549 (18)	−0.0093 (2)
Cl1	0.0177 (4)	0.0183 (4)	0.0164 (4)	0.0011 (3)	0.0005 (3)	−0.0003 (3)
Cl2	0.0123 (4)	0.0327 (5)	0.0309 (5)	−0.0007 (4)	0.0013 (3)	0.0069 (4)
S3	0.0108 (3)	0.0163 (4)	0.0139 (4)	0.0007 (3)	−0.0005 (3)	−0.0006 (3)
S4	0.0119 (4)	0.0297 (5)	0.0202 (4)	0.0024 (3)	−0.0022 (3)	−0.0077 (4)
S2	0.0102 (4)	0.0267 (5)	0.0223 (5)	0.0015 (3)	0.0006 (3)	0.0057 (4)
S1	0.0160 (4)	0.0135 (4)	0.0266 (5)	0.0012 (3)	−0.0077 (4)	−0.0010 (3)
C4	0.0162 (16)	0.0191 (18)	0.0251 (19)	0.0030 (14)	−0.0043 (14)	−0.0062 (15)
C3	0.0121 (15)	0.0167 (17)	0.0156 (17)	−0.0031 (13)	−0.0002 (13)	−0.0031 (13)
C1	0.0169 (16)	0.0179 (18)	0.0200 (18)	−0.0008 (14)	0.0007 (14)	0.0027 (14)
C2	0.0138 (15)	0.0233 (19)	0.0166 (17)	0.0016 (14)	−0.0019 (13)	−0.0019 (14)
N3	0.0121 (14)	0.042 (2)	0.0262 (19)	−0.0001 (14)	0.0008 (13)	0.0125 (16)
N1	0.0277 (17)	0.0149 (17)	0.039 (2)	0.0035 (14)	−0.0143 (15)	−0.0072 (15)
N7	0.0121 (14)	0.051 (2)	0.0263 (19)	−0.0026 (15)	0.0018 (14)	−0.0180 (17)
N2	0.0181 (15)	0.0173 (16)	0.0314 (18)	0.0034 (13)	−0.0036 (13)	−0.0054 (15)
N4	0.0114 (14)	0.041 (2)	0.0249 (18)	−0.0017 (14)	−0.0019 (13)	0.0098 (15)
N6	0.0153 (14)	0.0362 (19)	0.0166 (16)	−0.0091 (14)	0.0006 (13)	−0.0030 (15)
N5	0.0152 (15)	0.0377 (19)	0.0123 (15)	−0.0063 (14)	−0.0001 (12)	−0.0044 (13)
N8	0.0185 (16)	0.046 (2)	0.0237 (18)	−0.0033 (16)	−0.0016 (14)	−0.0193 (16)

### Geometric parameters (Å, º)

**Table:**

Cu1—S1	2.3091 (9)	C1—N2	1.320 (4)
Cu1—S2	2.2617 (10)	C2—N4	1.314 (5)
Cu1—S3	2.2728 (10)	C2—N3	1.327 (4)
Cu1—Cl2	3.1394 (12)	N3—H3A	0.867 (19)
Cu2—S1^i^	2.3081 (9)	N3—H3B	0.853 (19)
Cu2—S3	2.2747 (9)	N1—H1B	0.862 (19)
Cu2—S4	2.2421 (9)	N1—H1A	0.855 (19)
Cu2—Cl1	2.8687 (9)	N7—H7A	0.854 (19)
Cu1—Cu2	2.9470 (7)	N7—H7B	0.867 (19)
S3—C3	1.761 (3)	N2—H2B	0.852 (19)
S4—C4	1.717 (4)	N2—H2A	0.867 (19)
S2—C2	1.725 (4)	N4—H4A	0.852 (19)
S1—C1	1.739 (4)	N4—H4B	0.837 (19)
S1—Cu2^ii^	2.3081 (9)	N6—H6A	0.866 (19)
C4—N8	1.324 (5)	N6—H6B	0.857 (19)
C4—N7	1.325 (4)	N5—H5A	0.862 (19)
C3—N5	1.305 (4)	N5—H5B	0.856 (19)
C3—N6	1.320 (4)	N8—H8A	0.856 (19)
C1—N1	1.319 (5)	N8—H8B	0.852 (19)
			
Cl2—Cu1—S1	103.79 (3)	N5—C3—S3	119.8 (2)
Cl2—Cu1—S2	89.13 (4)	N6—C3—S3	119.1 (3)
Cl2—Cu1—S3	93.98 (3)	N1—C1—N2	119.7 (3)
S1—Cu1—S2	116.46 (4)	N1—C1—S1	120.1 (3)
S1—Cu1—S3	113.38 (4)	N2—C1—S1	120.1 (3)
S2—Cu1—S3	127.63 (4)	N4—C2—N3	117.5 (3)
Cl1—Cu2—S3	97.61 (3)	N4—C2—S2	119.7 (3)
Cl1—Cu2—S4	106.33 (3)	N3—C2—S2	122.8 (3)
Cl1—Cu2—S1^i^	86.56 (3)	C2—N3—H3A	120 (3)
S2—Cu1—S3	127.63 (4)	C2—N3—H3B	117 (3)
S2—Cu1—S1	116.46 (4)	H3A—N3—H3B	123 (4)
S3—Cu1—S1	113.38 (4)	C1—N1—H1B	113 (3)
S2—Cu1—Cu2	99.70 (3)	C1—N1—H1A	122 (3)
S3—Cu1—Cu2	49.63 (2)	H1B—N1—H1A	122 (4)
S1—Cu1—Cu2	107.25 (3)	C4—N7—H7A	118 (3)
S4—Cu2—S3	118.44 (3)	C4—N7—H7B	117 (3)
S4—Cu2—S1^i^	121.18 (4)	H7A—N7—H7B	125 (4)
S3—Cu2—S1^i^	116.02 (4)	C1—N2—H2B	118 (3)
S4—Cu2—Cu1	98.66 (3)	C1—N2—H2A	118 (3)
S3—Cu2—Cu1	49.58 (3)	H2B—N2—H2A	124 (4)
S1^i^—Cu2—Cu1	99.90 (3)	C2—N4—H4A	117 (3)
C3—S3—Cu1	105.83 (12)	C2—N4—H4B	123 (3)
C3—S3—Cu2	103.14 (11)	H4A—N4—H4B	120 (4)
Cu1—S3—Cu2	80.79 (3)	C3—N6—H6A	119 (3)
C4—S4—Cu2	107.36 (12)	C3—N6—H6B	118 (3)
C2—S2—Cu1	105.36 (12)	H6A—N6—H6B	122 (4)
C1—S1—Cu2^ii^	110.00 (12)	C3—N5—H5A	121 (3)
C1—S1—Cu1	110.01 (12)	C3—N5—H5B	122 (3)
Cu2^ii^—S1—Cu1	138.40 (4)	H5A—N5—H5B	117 (4)
N8—C4—N7	117.9 (4)	C4—N8—H8A	122 (3)
N8—C4—S4	119.4 (3)	C4—N8—H8B	117 (3)
N7—C4—S4	122.7 (3)	H8A—N8—H8B	117 (5)
N5—C3—N6	121.0 (3)		

Symmetry codes: (i) *x*−1, *y*, *z*; (ii) *x*+1, *y*, *z*.

### Hydrogen-bond geometry (Å, º)

**Table:**

*D*—H···*A*	*D*—H	H···*A*	*D*···*A*	*D*—H···*A*
N1—H1*A*···Cl1^iii^	0.85 (3)	2.44 (3)	3.230 (3)	154 (4)
N1—H1*B*···S4^ii^	0.87 (3)	2.55 (3)	3.404 (4)	171 (3)
N2—H2*A*···S2	0.87 (3)	2.54 (3)	3.379 (3)	164 (3)
N2—H2*B*···Cl1^iii^	0.86 (3)	2.56 (3)	3.344 (3)	153 (3)
N3—H3*A*···S1	0.87 (3)	2.65 (3)	3.488 (4)	164 (3)
N3—H3*B*···Cl2^iv^	0.86 (4)	2.57 (4)	3.373 (4)	156 (4)
N4—H4*A*···Cl2^iv^	0.85 (3)	2.49 (3)	3.309 (3)	161 (3)
N4—H4*B*···Cl2^v^	0.84 (3)	2.55 (3)	3.340 (3)	157 (4)
N5—H5*A*···Cl2	0.86 (3)	2.35 (3)	3.197 (3)	167 (4)
N5—H5*B*···Cl2^vi^	0.86 (3)	2.55 (4)	3.356 (3)	157 (3)
N6—H6*A*···Cl2^i^	0.87 (4)	2.66 (4)	3.323 (3)	134 (4)
N6—H6*B*···Cl1	0.86 (2)	2.44 (2)	3.296 (3)	174 (3)
N7—H7*A*···Cl1	0.85 (3)	2.61 (4)	3.343 (4)	145 (3)
N7—H7*B*···Cl1^vii^	0.87 (4)	2.55 (4)	3.367 (4)	157 (3)
N8—H8*A*···Cl1^vii^	0.86 (4)	2.54 (4)	3.326 (4)	152 (3)
N8—H8*B*···Cl1^viii^	0.85 (3)	2.55 (3)	3.298 (4)	148 (4)

Symmetry codes: (i) *x*−1, *y*, *z*; (ii) *x*+1, *y*, *z*; (iii) *x*+1, *y*+1, *z*; (iv) −*x*+1, −*y*+1, −*z*; (v) −*x*, −*y*+1, −*z*; (vi) −*x*, −*y*, −*z*; (vii) −*x*−1, *y*+1/2, −*z*+1/2; (viii) −*x*, *y*+1/2, −*z*+1/2.
